# Health Systems’ Resilience: COVID-19 Response in Trinidad and Tobago

**DOI:** 10.4269/ajtmh.20-0561

**Published:** 2020-06-09

**Authors:** Shelly-Ann Hunte, Karen Pierre, Roseann St. Rose, Donald T. Simeon

**Affiliations:** 1 Caribbean Centre for Health Systems Research and Development, The University of the West Indies, St. Augustine, Trinidad and Tobago;; 2 The University of Trinidad and Tobago, Arima, Trinidad and Tobago

## Abstract

Trinidad and Tobago, a small island developing state, has been ranked as number one in a report published by the University of Oxford that assessed responses to COVID-19 based on four of the six WHO criteria for rolling back COVID-19 “lockdown” measures. The key mitigation and containment strategies implemented by the country were evidence-informed and demonstrated an “all-of-government” approach. The COVID-19 health system response of this country demonstrates that although developing countries face many health system challenges, political will, evidence-informed decision-making, respect for science, and timely, coordinated, collaborative actions can strengthen the resilience and response of the health system during a health emergency.

## INTRODUCTION

Health systems in developing countries face similar challenges, including financial and human resource constraints, limited institutional capacity, poor infrastructure, nonexistent or weak health information systems, systemic health inequities, and a lack of transparency and accountability.^1^ These challenges compromise resilience and responsiveness during a health crisis, as a sudden demand for essential services can overburden the health systems and their institutions, resulting in disruption in service delivery, shortages in medical supplies, and overburdened healthcare workers.^2^

On March 12, 2020, the Ministry of Health of Trinidad and Tobago (T&T) reported the country’s first COVID-19 case.^3^ On May 28, the daily update was as follows: 116 (8.5/100,000) cases, 108 recovered, and eight (0.6/100,000) deaths^4^; at that time, no new cases had been reported since April 27. However, on May 30, an additional imported case was reported by the Ministry of Health.^5^

The twin-island Republic of T&T is classified as a developing country and a small island developing state by the United Nations.^6^ The country has a population of 1,363,985,^7^ and the health system includes both public and private sectors. Health services in the public sector are free to nationals at the point of access, whereas the private sector operates on a fee for service basis.^8^

Trinidad and Tobago’s response to the COVID-19 pandemic resulted in the country being ranked number one in a report published by the University of Oxford on May 1, 2020. The Oxford COVID-19 Government Response Tracker (OxCGRT) assessed countries based on four of the six WHO criteria for rolling back “lockdown” measures^9^: transmission controlled, test/trace/isolate, manage risk of exporting and importing cases, and community fully engaged. The OxCGRT’s ranking has resulted in many commentaries on the relatively successful T&T response, but it is also appreciated that containment of a respiratory virus pandemic may be easier in island states than elsewhere. This article is based on our review of information publicly shared by national officials. The assessment is presented in relation to WHO health systems functions.

### Leadership and governance.

Although the original consideration of the importance of leadership and governance relates specifically to health systems, T&T displayed leadership competence and capacity at both the national and sector levels. The country’s Prime Minister led the charge with senior health officials, and they engaged early, regularly, and clearly with the general population. Regular media briefings were aired on all local television channels, as well as multiple radio stations. Videos and media releases were made available on multiple Ministry websites and social media platforms.

Notably, the health system leadership and governance response began with the development and activation of the Pandemic COVID Plan and the Health Emergency Operation Centre. The latter was led by the executive management team at the Ministry of Health. All plans were aligned to the existing National Response Framework that embodies an all-hazards approach. The government also created a Cabinet-approved inter-ministerial task force to ensure a collaborative approach for the management of the COVID-19 pandemic. Last, a Cabinet-approved post-COVID road map to recovery task force was convened with a mandate to “build back better”*.*^10^

Key mitigation and containment strategies reflect the “all-of-government” approach. These include data-driven, evidenced-informed decision-making and guidelines from the Ministry of Health; the provision of financial resources from the Ministry of Finance; border/immigration restrictions implemented by the Ministry of National Security; remote work and pandemic leave policies prepared by the Ministry of Labor and Small Enterprise Development; the provision of social support services by the Ministry of Social Development and Family Services; and communications coordinated by the Ministry of Communications.

### Service delivery.

One of the early containment strategies implemented and communicated to the public was the establishment of a parallel health system, that is, a separate system to manage and treat confirmed COVID-19 cases, independent of the routine public health system.

Screening was conducted at points of entry at all health facilities, and COVID-suspected cases were isolated, diagnosed, and managed via the parallel health system. The latter created independent and increased capacity within the healthcare system, while at the same time mitigating excess risk to the routine system, including patients and workers. This system was designed to address the treatment needs of COVID-19 patients.

Requisite protocols were developed, updated, and communicated to healthcare workers and the public on quarantine, diagnosis, management, and treatment of COVID-19–suspected and –confirmed cases. Designated facilities for testing, diagnosis, and treatment were identified and communicated. At the time of this writing, there were 12 health facilities with more than 900 beds retrofitted to deliver different levels of care: severe/chronic care, intermediate care, step-down, and quarantine. Based on the country’s demographic and COVID-19 profile, the Ministry of Health calculated the country’s critical additional needs for COVID-19 (beds, ventilators, personal protective equipment [PPE], and other supplies).

The Ministry of Health led the expansion of laboratory testing, contact tracing, and risk assessment. A phased development of national capacity was used to increase testing sites, Dedicated COVID-19 hotlines were established to address queries from the public and provide initial screening and early guidance on safely accessing health care.

### Health financing.

The Ministry of Finance identified and the Cabinet approved increased budgetary allocations to various levels of the health sector, the Ministry of Health, Regional Health Authorities (RHAs), and the main procurement agency, the National Insurance Property Development Company Limited. There was also an assurance that additional requests for funding related to the pandemic would be considered and prioritized by the Ministry of Finance. As a public health measure, all persons (including nonnationals) were able to access free COVID-19–related health services in the parallel health system. There were therefore no direct financial barriers to care*.*

### Health workforce.

The T&T public healthcare sector, as in many other countries, has several human resource challenges. The main challenges relate to absolute shortages in the number, skill set, and distribution of healthcare workers.^8,11^ Specific to the pandemic, the projected need for additional staff was assessed, and measures were taken to address them, including invitation of retired health professionals to express their interest and availability to be contracted should there be a surge of COVID-19 cases, and addressing the shortage of intensive care unit (ICU) nurses by a special arrangement with the University of the West Indies for the training of local nurses and recruitment of ICU nurses from Cuba. The protection of frontline personnel was paramount, and they were provided with adequate and appropriate PPE, as well as quarantine facilities before returning to their families after working with COVID-19 patients. The Ministry also ensured psychological support was available for staff.

### Medical products, vaccines, and technology.

The Ministry of Health implemented several strategies that maintained adequate supplies for the routine health system, as well as ensured that needs specific to an effective COVID-19 response were met. The Ministry and RHAs were also supported in procuring supplies through its regional and international partners, i.e. the Pan American Health Organization and the United Nations Development Programme. In addition, several local manufacturers and other agencies bolstered availability with the local production of supplies for healthcare workers, including hand sanitizers, face shields, and face masks. The Ministry articulated a clear appreciation of increased and new needs related to specific items such as PPE and ventilators.

### Health information system.

Under the parallel health system, the routine process for collecting, collating, and storing health information was maintained. This system adequately addressed health information needs for COVID-19–related patient care, treatment, and surveillance, inclusive of contact tracing.

### Limitations of the T&T approach.

An acceleration of training and validation of local laboratories was lacking and would have improved the country’s ability to scale up its testing capacity. This was exacerbated by the absence of a national public health laboratory capable of conducting the required testing. The T&T health system’s response also lacked inclusion of media and local influencers in the relevant COVID-19 committees or task forces. This inclusion would have bolstered public trust in the data reported, as well as their acceptance of reduced risk of contracting COVID-19 when accessing the routine healthcare system. In addition, there has been no evidence on how well the routine healthcare system has been working.

## CONCLUSION

Ensuring that key mitigation and containment strategies were well-coordinated, collaborative, evidence-informed, and timely was critical to the success of the T&T health system response to COVID-19. This was especially so for the leadership and governance and service delivery functions*.* The country’s health system response is a reminder that even in developing countries, fraught with many health system challenges, a combination of political will, decisiveness, respect for science, and the utilization of evidence-informed policies can have positive outcomes for populations during a health crisis. As the country rolls out its phased reopening, including the reopening of its borders, the expectation is that the underpinning principles and actions that contributed to the initial successful containment of COVID-19 will be sustained.
